# A multitarget approach to drug discovery inhibiting *Mycobacterium tuberculosis* PyrG and PanK

**DOI:** 10.1038/s41598-018-21614-4

**Published:** 2018-02-16

**Authors:** Laurent R. Chiarelli, Giorgia Mori, Beatrice Silvia Orena, Marta Esposito, Thomas Lane, Ana Luisa de Jesus Lopes Ribeiro, Giulia Degiacomi, Júlia Zemanová, Sára Szádocka, Stanislav Huszár, Zuzana Palčeková, Marcello Manfredi, Fabio Gosetti, Joël Lelièvre, Lluis Ballell, Elena Kazakova, Vadim Makarov, Emilio Marengo, Katarina Mikusova, Stewart T. Cole, Giovanna Riccardi, Sean Ekins, Maria Rosalia Pasca

**Affiliations:** 1 Department of Biology and Biotechnology “Lazzaro Spallanzani”, University of Pavia, Pavia, Italy; 2 Collaborations Pharmaceuticals, Inc., 840 Main Campus Drive, Lab 3510, Raleigh, North Carolina 27606 USA; 3 Molecular and Cellular Biophysics Program, Department of Biochemistry and Biophysics, University of North Carolina, Chapel Hill, NC 27599 USA; 4 Centro de Biologia Molecular “Severo Ochoa”, Universidad Autónoma de Madrid, Madrid, Spain; 5 Department of Biochemistry, Faculty of Natural Sciences, Comenius University in Bratislava, Bratislava, Slovakia; 6 Department of Sciences and Technological Innovation, University of Piemonte Orientale, Alessandria, Italy; 7 Diseases of the Developing World, GlaxoSmithKline, Tres Cantos, Madrid, Spain; 8 Lab for Biomedicinal Chemistry, Bach Institute of Biochemistry, Research Center of Biotechnology of the Russian Academy of Sciences, Moscow, 119071 Russia; 9 Global Health Institute, Ecole Polytechnique Fédérale de Lausanne (EPFL), Lausanne, CH-1015 Switzerland; 10 Collaborative Drug Discovery, Inc., Burlingame, CA USA

## Abstract

*Mycobacterium tuberculosis*, the etiological agent of the infectious disease tuberculosis, kills approximately 1.5 million people annually, while the spread of multidrug-resistant strains is of great global concern. Thus, continuous efforts to identify new antitubercular drugs as well as novel targets are crucial. Recently, two prodrugs activated by the monooxygenase EthA, 7947882 and 7904688, which target the CTP synthetase PyrG, were identified and characterized. In this work, microbiological, biochemical, and *in silico* methodologies were used to demonstrate that both prodrugs possess a second target, the pantothenate kinase PanK. This enzyme is involved in coenzyme A biosynthesis, an essential pathway for *M. tuberculosis* growth. Moreover, compound 11426026, the active metabolite of 7947882, was demonstrated to directly inhibit PanK, as well. In an independent screen of a compound library against PyrG, two additional inhibitors were also found to be active against PanK. In conclusion, these direct PyrG and PanK inhibitors can be considered as leads for multitarget antitubercular drugs and these two enzymes could be employed as a “double-tool” in order to find additional hit compounds.

## Introduction

Antibiotic resistance remains a considerable problem for tuberculosis (TB) treatment, despite the introduction of new antitubercular drugs into therapy^1^. In 2015, an estimated 480,000 new cases of multidrug-resistant TB (MDR-TB) and an additional 100,000 people with rifampicin-resistant TB were documented with high mortality rates, particularly in India, China and the Russian Federation^2^. This highlights the urgent need for new antitubercular drugs with a novel mechanism of action in order to address drug-resistant TB.

Many antitubercular compounds only inhibit a single target and are thus more effective when combined with other agents, in a combination therapy^3,4^. This therapy is exemplified by the widely used DOTS regimen comprising the following four antitubercular compounds: isoniazid, rifampicin, ethambutol, and pyrazinamide. Combination therapy should prevent the emergence of drug-resistant isolates of *Mycobacterium tuberculosis* when compared with monotherapy^2^. Considering these aspects, there is growing interest in the identification of antitubercular compounds that hit multiple targets. In “multitargeting” therapy, a single drug has more than one target as exemplified by the ethylene diamine drug, SQ109, an uncoupler which inhibits two distinct proteins involved in cell wall and menaquinone biosynthesis^4^. Multitargeting compounds can be divided into three different groups: 1. The targets belong to the same metabolic pathway (series inhibition); 2. The targets are unrelated, but an inhibitor could affect a common substrate (parallel inhibition); 3. The targets are present in series and/or in parallel (network inhibition)^4–6^. In this context, a new compound targeting novel *M. tuberculosis* enzymes appears to be an ideal candidate.

**Figure 1:**
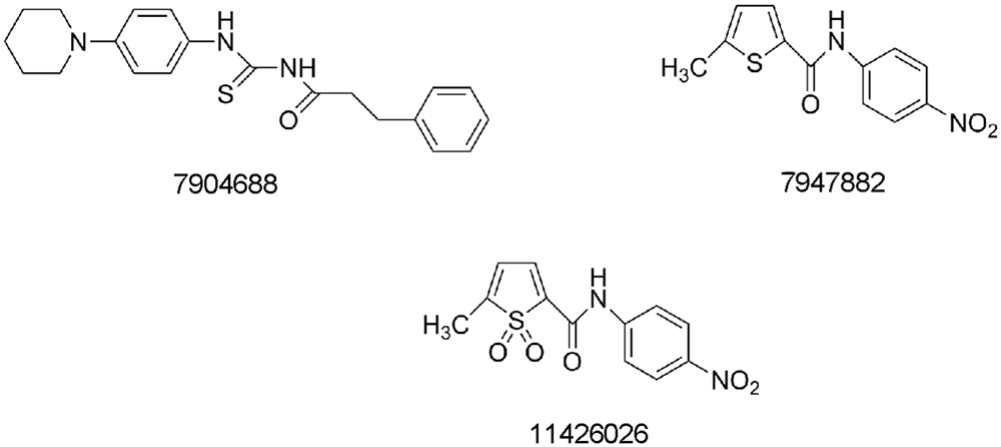
The two prodrugs 7904688 and 797882 as well as 11426026, which is the active metabolite of 797882.

Recently, it was demonstrated that two antitubercular compounds 7947882 (5-methyl-N-(4-nitrophenyl)thiophene-2-carboxamide) and 7904688 (3-phenyl-N-[(4-piperidin-1-ylphenyl)carbamothioyl]propanamide) are both prodrugs activated by the EthA monooxygenase that inhibit the activity of the CTP synthetase, PyrG, a novel *M. tuberculosis* drug target. Moreover, the active metabolite of 7947882, 11426026, was identified and characterized as a PyrG inhibitor, too (Fig. 1)^7^. PyrG was used for target-based screening of antitubercular compound libraries including the GlaxoSmithKline antimycobacterial compound set (GSK TB-set)^8^ leading to the discovery of a series of 4-(pyridin-2-yl)thiazole derivatives as PyrG inhibitors^9^. However, except for one compound, these inhibitors also efficiently targeted the human CTP synthetase^9^.

In this work, using microbiological, biochemical and *in silico* approaches, we continued the characterization of compounds 7947882 and 7904688 and identified a second cellular target, the *M. tuberculosis* pantothenate kinase (PanK), encoded by the *coaA* gene^10,11^. PanK catalyses the first and rate-limiting step in the Coenzyme A (CoA) biosynthetic pathway, where pantothenate (vitamin B5) is converted to 4′-phosphopantothenate, using ATP as a phosphate donor^10,11^. This pathway is very attractive as a source of novel drug targets because CoA is required both as an essential cofactor and for the regulation of key metabolic enzymes in numerous cellular pathways, such as the biosynthesis and the catabolism of lipids^11^. Moreover, *coaA* codes for the sole *M. tuberculosis* PanK enzyme and this is essential both for growth *in vitro* and *in vivo*^12^. The PanK crystal structure is available in the apo form or in complex with different inhibitors^13–15^. The PanK inhibitors identified until now belonged to two classes: 1. Compounds binding to the pantothenate binding site of the enzyme; 2. Compounds interfering with ATP binding. Unfortunately, these PanK inhibitors were either inactive or poorly active against *M. tuberculosis* growth *in vitro* probably because of poor bioavailability^14,15^.

Further, in this work, by biochemical and *in silico* approaches, we identified some new inhibitors of both *M. tuberculosis* PyrG and PanK enzymes. This finding paves the way for a multitargeting hit compound discovery process, using these two enzymes, in order to develop new antitubercular drugs.

## Results

### Isolation and characterization of new *M. tuberculosis* mutants resistant to 7947882 and 7904688

In our previous work, spontaneous mutants resistant to 7947882 and 7904688 were isolated and characterized, and shown to harbor mutations in the *ethA* (*rv3854c*) and/or *pyrG* (*rv1699*) genes, coding for the activator and the target of both compounds, respectively^7^. In order to better understand the mechanisms of action and resistance of these compounds, new resistant mutants of *M. tuberculosis* have been isolated, showing cross-resistance to both 7947882, 7904688 and to 11426026, the active metabolite of 7947882^7^ (Table 1) (frequency: 2 × 10^−8^). To find the mutation(s) responsible for resistance, the new isolated resistant mutants 82.21, 88.1 and 88.2 were chosen for Illumina whole genome sequencing (Table 1). *M. tuberculosis* 88.1 and 88.2 mutants revealed only a mutation in the *coaA* gene, coding for the pantothenate kinase (PanK), which catalyses the first step in the Coenzyme-A biosynthesis^11^. Moreover, in addition to the A620G mutation in *coaA* gene, the 82.21 mutant harbored a second mutation in *ethA*. Finally, the other six *M. tuberculosis* mutants (88.3, 88.4, 88.5, 88.9, 88.11, 88.12), that were screened for possible mutations in the *coaA*, *pyrG*, and *ethA* genes, all contained only the same mutation in *coaA* gene (Table 1).

**Table 1:** Main features of new *M. tuberculosis* mutants resistant to 7947882 and 7904688.

*M. tuberculosis* strains	MIC (µg/ml)				Mutations (aa change)	
	7947882	7904688	11426026	Ethionamide	*ethA*	*coaA*
H37Rv	0.5	0.5	1	1	—	—
82.21	>40	>40	2.5	10	Δ1283–4	A620G (Q207R)
88.1	2.5	10	2.5	1	—	A620G (Q207R)
88.2	2.5	10	2.5	1	—	A620G (Q207R)
88.3, 88.4, 88.5, 88.9, 88.11, 88.12	2.5	10	2.5	1	—	A620G (Q207R)
81.10*	>40	>40	1	10	Δ1109–1137	—

*Laboratory collection.

These results suggested an additional role for PanK in the mechanism of resistance to these compounds, probably as a second target.

### Biochemical validation of PanK as target of 7947882 and 7904688

To demonstrate if the activated prodrugs and the 11426026 metabolite were able to inhibit PanK, the recombinant wild-type and the Q207R mutant *M. tuberculosis* enzymes were produced in *Escherichia coli* and characterized (Table S1). The PanK mutant (Q207R) enzyme showed impaired kinetic parameters, particularly regarding the *K*_m_ value for ATP, which was nearly 20-fold higher than that of the wild-type (Table S1). These results suggested that the ATP-binding site of PanK could be involved in the binding to the inhibitors. In fact, a similar situation was found for the PyrG V186G mutant, for which the alteration in the ATP-binding site was responsible for the resistance to 7947882 or 7904688 activated compounds^7^ (Table S1).

**Figure 2:**
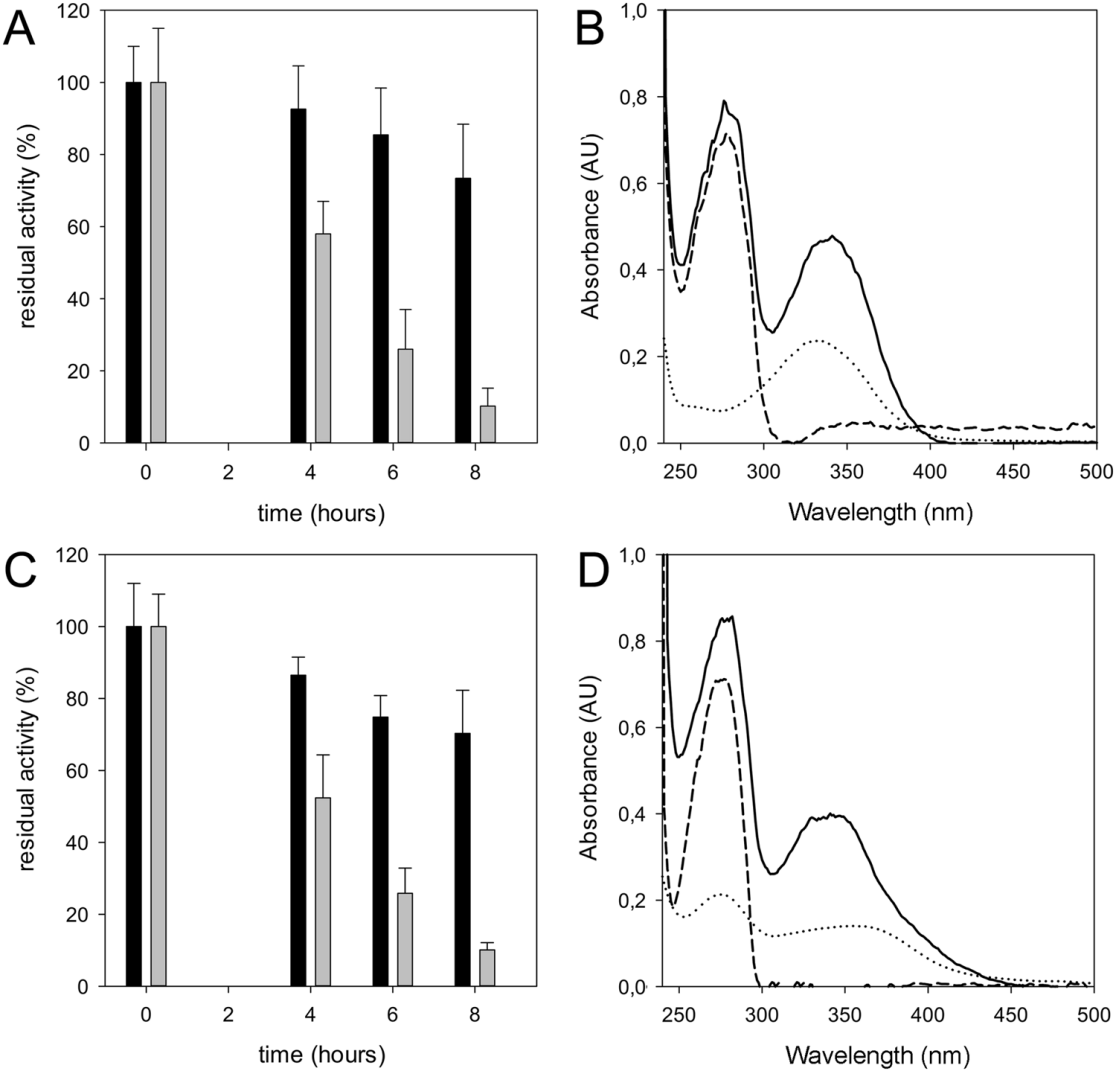
Inhibition of PanK activity by the EthA-activated 7947882 and 7904688 compounds. (**A**) Inhibition of PanK activity during the incubation with 7947882 and EthA. Black bars represent the activities of the control without NADPH, whilst grey bars represent the residual activities of the full EthA reaction. (**B**) UV-visible spectra of the re-purified PanK after incubation with 7947882 and EthA. Solid line: PanK from the full EthA reaction; dashed line: PanK from blank reaction; dotted line: spectrum of 7947882 at 20 μM. (**C**) Inhibition of PanK activity during the incubation with 7904688 and EthA. Black bars represent the activities of the control without NADPH, whilst grey bars represent the residual activities of the full EthA reaction. (**D**) UV-visible spectra of the re-purified PanK after incubation with 7904688 and EthA. Solid line: PanK from the full EthA reaction; dashed line: PanK from blank reaction; dotted line: spectrum of the 7904688 at 20 μM.

To demonstrate the effectiveness of the active metabolites of the two prodrugs on PanK, their effects upon EthA activation were assessed. Each compound was incubated in the presence of EthA and PanK and the residual activity of the latter enzyme was checked during the reaction. In the blank controls, NADPH was omitted from the reaction mixture, to avoid the EthA-catalysed reaction. Under these conditions, PanK retained more than 80% of its activity after 8 hours of incubation. On the contrary, when NADPH was added, PanK lost more than 90% of its activity within the same time (in the presence of either 7947882 or 7904688) (Fig. 2), confirming that the EthA-activated compounds are also able to inhibit PanK.

The binding of the activated prodrugs to PanK was further investigated by examining the UV-visible spectra of the re-purified enzyme. In particular, the reaction samples were characterized by an additional peak between 300 and 400 nm, characteristic of the compounds but absent in the spectra of the blank reaction (Fig. 2). These results underlined that the active metabolites of both prodrugs are able to bind PanK, like PyrG.

**Figure 3:**
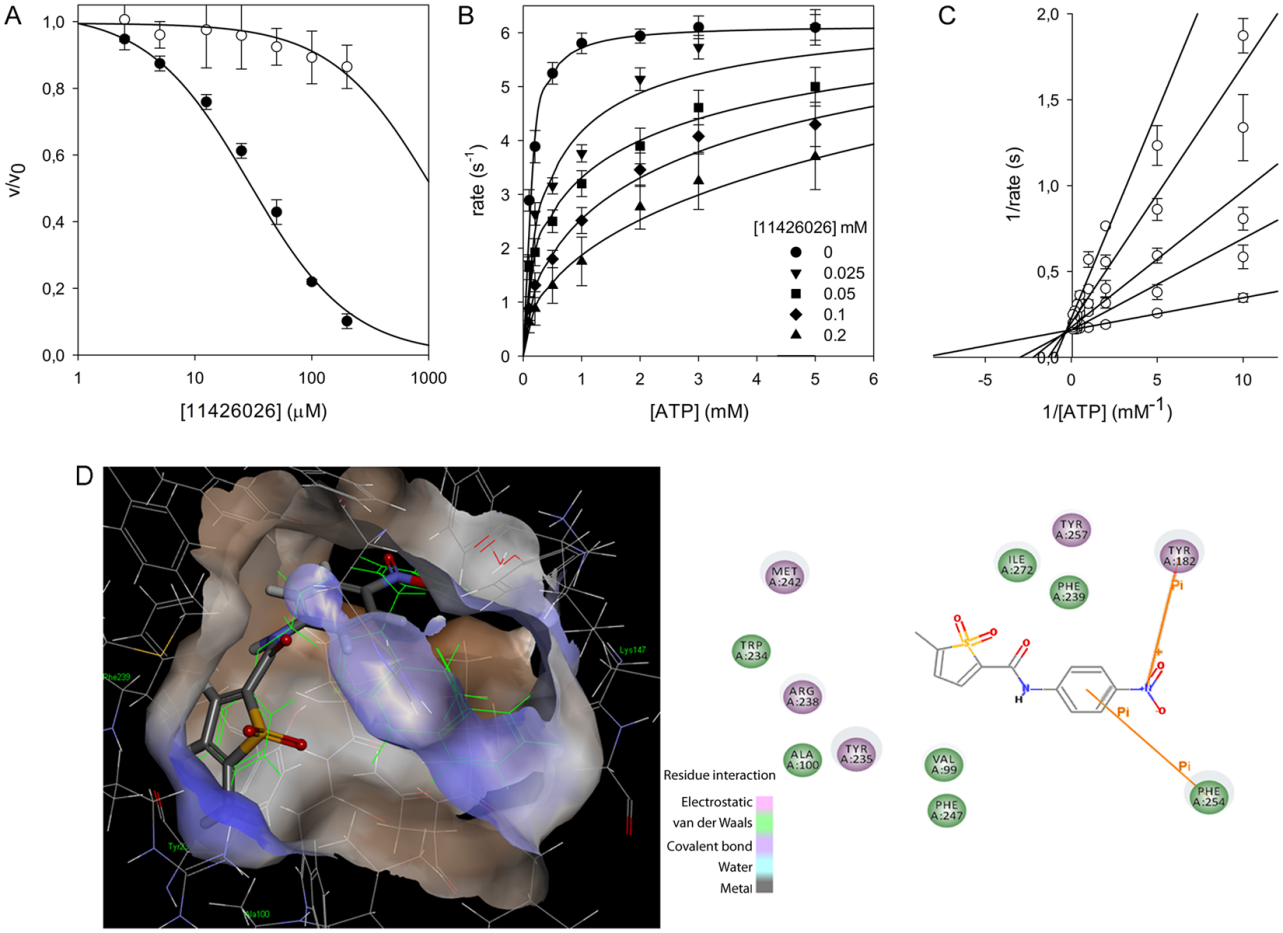
Inhibition of PanK activity by 11426026 metabolite. (**A**) IC_50_ determination for 11426026 against wild type (closed symbol) and Q207R mutant (open symbol) PanK. IC_50_ values were determined at concentrations of ATP corresponding to the *K*_m_ values for each enzyme (0.19 mM for the wild-type protein and 3.5 mM for the mutant one), by fitting the experimental data as reported in Methods. (**B**) Steady state kinetics analysis towards ATP of PanK in the presence of different concentrations of 11426026 compound. (**C**) Global reciprocal plot of data in panel B. (**D**) 11426026 Docked in PanK structure (showing ATP binding site volume and triazine compound in green) and 2D interaction plot.

In order to better analyse the role of PanK as a second target, the direct effects of the previously identified 11426026 active metabolite against wild type and mutant enzyme were then evaluated. This confirmed that the wild type PanK enzyme was effectively inhibited by the compound (IC_50_ = 29.3 ± 2.0 μM), whilst the Q207R mutant was practically insensitive (IC_50_ >1 mM) (Fig. 3). Moreover, the kinetic analysis of PanK in the presence of 11426026 demonstrated that the compound acts as a competitive inhibitor towards the ATP binding site, with a Ki of 22.9 ± 1.3 μM (Fig. 3).

In order to study in depth the binding of 11426026 to PanK, this metabolite was docked within the PanK crystallographic structure (PDB code 4BFW)^12^. From this *in silico* analysis using Discovery Studio, it has been shown that 11426026 might dock in the PanK ATP-binding site (Libdock score 82.99) (Fig. 3). The phenyl ring of 11426026 appears to Pi stack with Phe254, while the nitro group may interact with Tyr182. The metabolite also partially overlaps the triazole ligand from crystal structure^14^. This is similar to our observation of its proposed binding in the ATP-binding site of the PyrG structure^7^.

These results confirmed that as for PyrG, the active metabolite 11426026, as well as the activated prodrugs, inhibit PanK by binding at its ATP binding site. Besides, these data account for the resistance to these compounds found in the *M. tuberculosis* mutants with a mutation in *coaA* gene (Table 1). In addition, further confirmation was provided by metabolic labelling of *M. tuberculosis* strain H37Ra with [14C] acetate that revealed severe inhibition of incorporation of the radiolabel into the lipids^7^, which also could point to interference with CoA metabolism (Figure S1). For this reason, the two prodrugs and 11426026 can be considered as three multitargeting compounds that affect both PyrG and PanK activity.

### Searching for multitargeting compounds inhibiting both PyrG and PanK

Taking into account our finding of new multitargeting compounds affecting PyrG and PanK, we wished to investigate if any of the previously identified PyrG inhibitors also had PanK as a second target. In our previous work, two different strategies were followed to identify further PyrG inhibitors:

1. *In silico* virtual substructure searching of the Collaborative Drug Discovery (CDD) database allowed us to find twelve potential ligands with known antitubercular activity. Four of these compounds were tested in the PyrG enzyme assay revealing only one as an effective PyrG inhibitor (CDD-823953)^7^ (Table 2).

**Table 2:** GSK and CDD compound structures with PyrG and PanK activities.

Compounds	Identifier	PyrG IC_50_ (μM)	PanK IC_50_ (μM)	MIC (μM)	LibDock score
	GSK1570606A	4.2	n. i.	9.3	104.59
	GSK735826A	22	70	2.7	94.69
	GSK920684A	22	n. i.	3.5	106.90
	CDD-934506	n. i.	40	0.87	121.81
	CDD-823953	80	250	4.39	116.15
2. Target-based screening of the GSK TB-set identified three active inhibitors in the enzyme assay (GSK1570606A, GSK920684A, GSK735826A)^9^ (Table 2).

Having demonstrated that PyrG and PanK are suitable for a multitargeting strategy as 11426026 was a common inhibitor of both enzymes, all PyrG inhibitors were also assayed for their ability to inhibit PanK.

**Figure 4:**
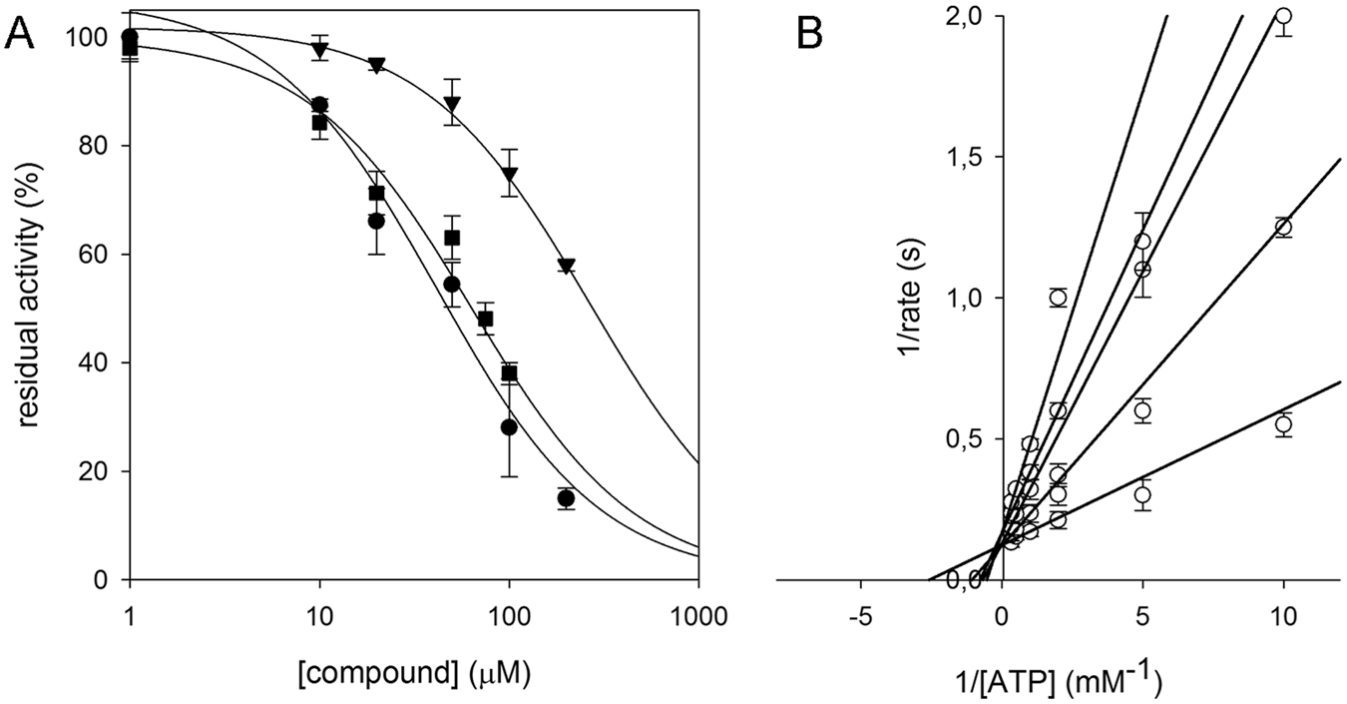
Identification of new PanK inhibitors. (**A**) IC_50_ determination against PanK for CDD-823953 (▼), CDD-934506 (●) and GSK-735826A (■). (**B**) Global reciprocal plot of steady state kinetics analysis towards ATP of PanK in the presence GSK-735826 compound, highlight the competitive nature of the inhibition.

**Figure 5:**
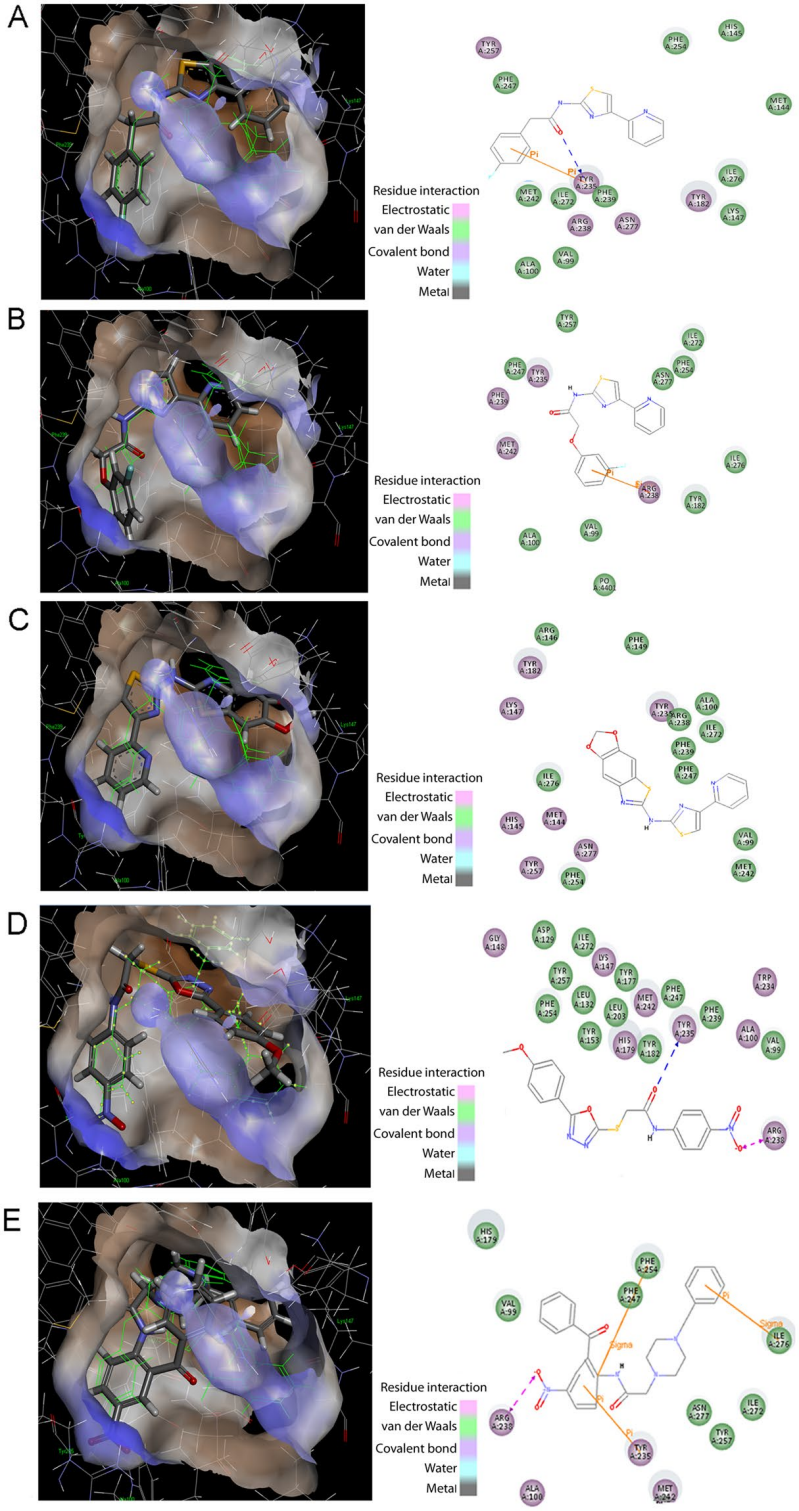
Docking of GSK and CDD compounds into the PanK crystal structure. (**A**) GSK1570606A, (**B**) GSK920684A, (**C**) GSK735826A, (**D**) CDD-934506, (**E**) CDD 823953 in ATP binding site with 2D interaction plots. All molecules are compared to the triazine ligand from the crystal structure shown as the green thin line.

From the CDD database, compound CDD-823953, active against PyrG, was also found to be a weak inhibitor of PanK (IC_50_ = 250 μM). In addition, another compound CDD-934506, which was inactive against PyrG, was shown to inhibit PanK moderately (IC_50_ = 40 μM) (Fig. 4; Table 2). Then, these two CDD compounds (CDD-823953 and CDD-934506) were successfully docked into the PanK active site, showing high Libdock scores (Fig. 5).

From the GSK TB-set, only one compound GSK735826A (out of three) was shown to be active against both PanK (IC_50_ = 70 μM) and PyrG (Fig. 4; Table 2). To better investigate the mechanism of action of this inhibitor (GSK735826A), a kinetic analysis of PanK in the presence of different concentrations of the compound was performed. As previously shown for PyrG, GSK735826A was found to act as a competitive inhibitor toward the ATP-binding site of PanK (Ki = 65.3 ± 4.33 μM) (Fig. 4). All three selected GSK inhibitors (GSK1570606A, GSK920684A, GSK735826A) were also docked in the PanK active site (Fig. 5). Interestingly, although the three GSK compounds showed a high score when docked in PanK, only one (GSK735826A) was active in the enzymatic assay (Fig. 4). This finding could be partly due to the different hydrogen-bond acceptors (K147, H179, R238, N277 and H280) in the ATP binding site, which could explain the diverse behaviour of some compounds against the two proteins.

The two common PyrG and PanK inhibitors, CDD-823953 and GSK735826A, both docked similarly to 11426026 in the ATP-binding site of PanK (Fig. 5).

Overall these data confirm that these two inhibitors are further examples of multitargeting compounds affecting PanK and PyrG activities.

## Discussion

The pantothenate kinase PanK is involved in coenzyme A (CoA) biosynthesis, an essential pathway for *M. tuberculosis* growth and the *M. tuberculosis* enzyme is significantly different from the human counterparts. For these reasons, PanK was considered a valuable potential drug target^16^. However, recent genetic studies demonstrated that this enzyme shows poor vulnerability, thus reducing its perceived value as a potential drug target^15^.

In this investigation, by microbiological, biochemical and *in silico* approaches, PanK was validated as an additional target of two antitubercular prodrugs (7947882 and 7904688), which primarily affect the *M. tuberculosis* CTP synthetase PyrG. It is conceivable that, despite its poor vulnerability, the inhibition of PanK could act synergistically with the inhibition of PyrG, thus increasing the activity of these compounds. It is noteworthy that in order to inhibit PyrG these two compounds needed to be activated by EthA monooxygenase, an enzyme known to be the activator of several antitubercular compounds, including ethionamide^17^. EthA activation of the compounds was also demonstrated to be necessary for the inhibition of the wild-type recombinant PanK, while the active metabolite of 7947882, 11426026, was directly active against PanK. This metabolite was then confirmed to affect both PyrG and PanK with a common mechanism of action, and thus it could represent an interesting scaffold for the future development of multitargeting compounds.

The *M. tuberculosis* CTP synthetase PyrG appears to be druggable because several inhibitors with antitubercular activity belonging to different chemical classes were identified^7,9^. As we found a common inhibitor of both the PyrG and PanK enzymes, it was reasonable to anticipate that other CTP synthetase inhibitors could inhibit PanK in turn. Consequently, all the PyrG inhibitors, which we had previously characterized, were assayed against PanK. Two compounds firstly identified as PyrG inhibitors CDD-823953 (from substructure searching the CDD database of antitubercular compounds) and GSK735826A (from screening of the GSK library of antitubercular compounds) were shown to affect pantothenate kinase activity as well. It is noteworthy that GSK735826A was not a potent PyrG inhibitor, having rather high IC_50_ against the CTP synthetase^9^. Indeed, metabolic labelling of *M. tuberculosis* cells with [14 C]uracil revealed that this compound exerts lower inhibition of CTP formation than the other two tested 4-(pyridin-2-yl)thiazoles^9^. Nevertheless, GSK735826A was demonstrated to impair lipid metabolism too and this was especially obvious after the longer drug treatment (Figure S1). This inhibition can be attributed to depletion of activated CDP-derivatives, necessary for the biosynthesis of phospholipids, but it could also be related to a decrease in CoA levels, thus explaining the similar effects exerted by all three GSK compounds^9^. Moreover, GSK735826A shows the best antitubercular activity among these three, suggesting a synergistic effect of the simultaneous inhibition of these two targets.

**Figure 6:**
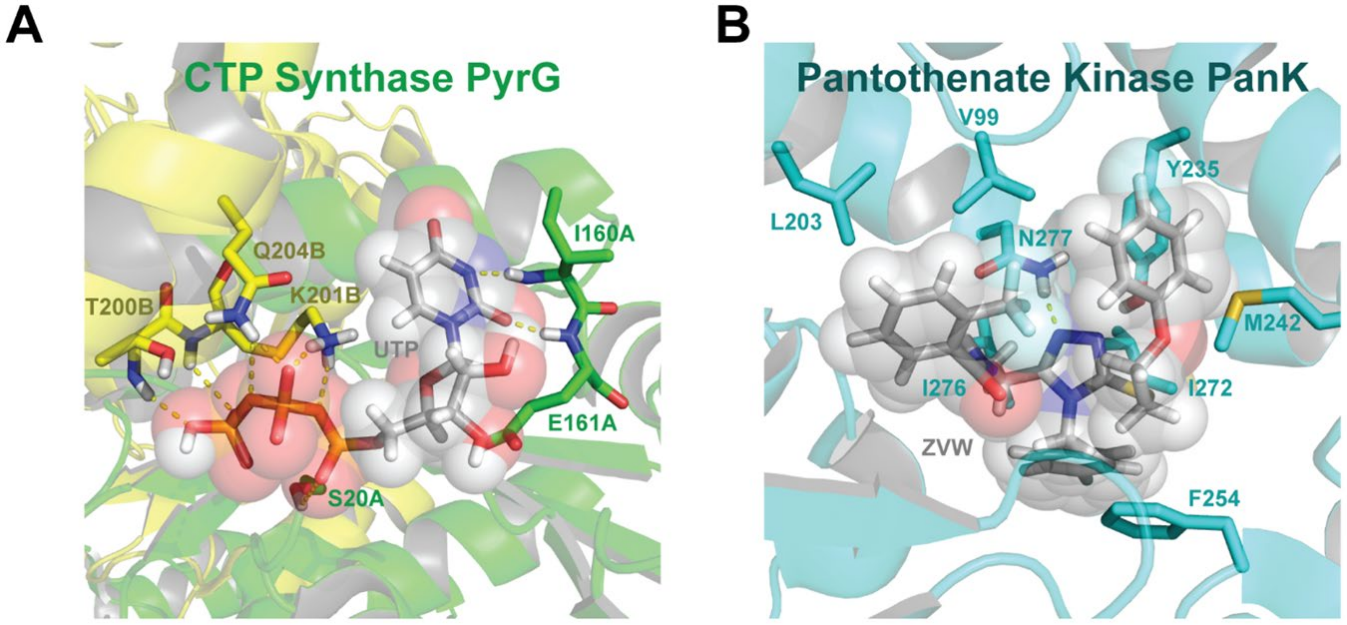
Cartoon representation of the ATP binding pockets of *M. tuberculosis* CTP Synthase PyrG (**A**, PDB:4ZDK) and Pantothenate Kinase PanK (**B**, PDB:4BFW) created with PyMOL (https://www.pymol.org/).

Our docking analysis of both PyrG and PanK crystal structures indicated a low degree of structural homology (10.8% similarity using the default parameters in blast and using needle; data not shown). In the PyrG structure (Fig. 6), UTP has multiple electrostatic crystal structure interactions with residues from two chains, A/B, including: (1) hydrogen-bonds between UTP and backbone amides of I160A, E161A, T200B, and Q204B; (2) hydrogen-bonding with side chains of S20A and Q204; (3) a salt-bridge between K201B and a phosphate group from UTP. In the PanK structure (Fig. 6), the binding between PanK and the triazine compound has both hydrophobic and electrostatic components. There are π-stacks between the aromatic rings of the triazine compound and F254/Y235, as well as a methionine-π interaction. Additionally, there is a hydrogen bond between the side chain of N277 and the triazine moiety. The remaining major interactions are hydrophobic, with the triazine compound having close proximity with V99, L203, I272, and I276.

While binding of the triazine compound and UTP are dissimilar, both of these pockets are known to bind ATP. The ATP-binding pocket of PanK also contains multiple hydrogen-bond acceptors (data not shown), including K147, H179, R238, N277 and H280 that could accommodate the negatively charged phosphate groups (Fig. 6). These differences might go some way to explain why only a few compounds have activity against both proteins.

Even if both enzymes are present in man, it is noteworthy that the *M. tuberculosis* PanK is significantly different from the human pantothenate kinases, both in terms of sequence homology and regulatory properties^14^. By contrast mycobacterial and mammalian CTP synthetases show significant similarity. Nevertheless, we previously produced the recombinant human enzyme, and demonstrated the possibility to identify inhibitors specific for the mycobacterial enzyme, such as 11426026. Notably, the prodrugs 7947882 and 7904688^7^, as well as the GSK compounds^8^, were previously demonstrated to possess a very low toxicity against human cell lines, thus confirming the possibility to inhibit the two enzymes without effects on the host.

In conclusion, we have demonstrated that two essential *M. tuberculosis* enzymes, PyrG and PanK share some common inhibitors, which paves the way for a “double targeting” approach to drug screening in order to identify more attractive compounds with potential as new antitubercular drugs.

## Methods

### Chemicals

7947882 [5-methyl-N-(4-nitrophenyl)-2-thiophenecarboxamide] and 7904688 [3-phenyl-N-({[4-(1-piperidinyl)phenyl]-amino} carbonothioyl) propanamide] compounds were purchased from ChemBridge Corporation (http://www.chembridge.com/index.php). CDD-815202 (3-iodo-4-methyl-N-(2-methyl-4-nitrophenyl)benzamide), CDD-934506 (2-((5-(4-methoxyphenyl)-1,3,4-oxadiazol-2-yl)sulfanyl)-N-(4-nitrophenyl)acetamide), CDD-833850 (5-chloro-2-hydroxy-N-(2-methoxy-4-nitrophenyl)benzamide), CDD-823953 (N-(2-benzoyl-4-nitrophenyl)-2-(4-benzylpiperazin-1-yl)acetamide), GSK1570606A (2-(4-fluorophenyl)-N-(4-(pyridin-2-yl)thiazol-2-yl)acetamide, GSK735826A N-(4-(pyridin-2-yl)thiazol-2-yl)-(1,3)dioxolo(4′,5′:4,5)benzo(1,2-d)thiazol-6-amine, and GSK920684A 2-(3-fluorophenoxy)-N-(4-(pyridin-2-yl)thiazol-2-yl)acetamide were from MolPort (Riga, Latvia). Phenylmethylsulfonylfluoride (PMSF), DNase, ATP, pantothenic acid, NADH, phospho(enol)pyruvate, pyruvate kinase/L-lactic dehydrogenase solution, isopropyl-β-D thiogalactopyranoside (IPTG), ampicillin, kanamycin and hygromicin were from Sigma-Aldrich. Other chemicals were reagent grade.

### Bacterial strains and growth conditions

Cloning steps were performed in *Escherichia coli* XL1-Blue, following standard methods^18^. *E. coli* cultures were grown either in Luria-Bertani (LB) broth or on LB agar. *M. tuberculosis* strains were grown aerobically at 37 °C either in Middlebrook 7H9 medium or on Middlebrook 7H11 agar, both supplemented with 10% OADC Middlebrook Enrichment. When necessary, antibiotics were added at the following concentrations: ampicillin, 100 µg/ml; kanamycin, 50 µg/ml.

All the experiments with *M. tuberculosis* H37Rv were performed in Biosafety level 3 laboratory by authorized and trained researchers.

### Isolation and characterization of *M. tuberculosis* mutants resistant to 7947882 and 7904688

The isolation of *M. tuberculosis* mutants was performed by plating ~10^10^ cells from an exponential growth phase culture of wild-type *M. tuberculosis* in 7H11 medium containing different concentrations of 7947882 and 7904688, ranging from 5 to 20-fold the MIC. Genomic DNA of resistant mutants was purified and sequenced by Illumina HiSeq 2000 technology at IGA Technology Services S.R.L. (Udine, Italy). For the bioinformatic analysis of Illumina data, the repetitive PE and PPE gene families were discarded as well as SNPs and Indels with less than 50% probability. The mutations found in *ethA* and/or *coaA* (*Rv1092c*) gene were confirmed by Sanger sequencing (Eurofins MWG Operon) (Table S2).

### Expression and purification of *M. tuberculosis* PanK enzyme

The *coaA* gene from *M. tuberculosis* H37Rv was amplified by standard PCR and PCR fragments were cloned in the pET28a vector, to give pET28a/*coaA* recombinant plasmid (Table S2). *M. tuberculosis* PanK wild type recombinant enzyme was produced fused with a His6 tag in *E. coli* BL21(DE3) cells grown in LB medium containing 50 µg/ml kanamycin by 12 hours of induction with 0.5 mM IPTG at 25 °C. Cells were resuspended in 25 mM sodium phosphate pH 8.0, 600 mM NaCl (Buffer A) containing 1 mM PMSF and 200 μg/ml DNase, disrupted by sonication, and centrifuged (30,000 g, 50 min). The supernatant was loaded onto a HisTrap crude column (1 ml, GE Healthcare), and washed with 100 mM imidazole in buffer A, then PanK elution was achieved with 250 mM imidazole in the same buffer. The eluted protein was dialyzed in 50 mM Tris-HCl pH 8.0, 150 mM NaCl, 5% glycerol, and stored at −80 °C. Sample purity was checked by SDS-PAGE and protein concentration was evaluated by absorbance at 280 nm (ε = 36900 M^−1^ cm^−1^). The PanK resistant mutant (Q207R) protein was obtained by site-directed mutagenesis on pET28a/*coaA* recombinant plasmid, using the Quik Change procedure (Agilent) using primers designed to include the desired mutation. The mutant enzyme was expressed and purified as well as the wild-type protein.

### Enzymatic activity assays, steady state kinetics and inhibition assays

PanK activity was determined using a continuous spectrophotometric pyruvate kinase/lactate dehydrogenase-coupled assay, measuring the decrease of NADH at 340 nm (ε = 6.22 mM^−1^ cm^−1^)^19^. Typically, the assays were performed at 37 °C in 50 mM HEPES pH 8.0, 10 mM MgCl_2_, 2 mM ATP, 2 mM pantothenic acid, 0.2 mM NADH, 0.5 mM phospho(enol)pyruvate, 5 units pyruvate kinase and lactate dehydrogenase, 0.5 μM PanK. The reaction was initiated by addition of ATP or pantothenic acid substrate.

Steady-state kinetic parameters were determined by assaying the enzymes at least at 8 different concentrations of their substrates. All experiments were performed in triplicate, and the kinetic constants, *K*_m_ and kcat, were determined fitting the data to the Michaelis-Menten equation using Origin 8 software. For IC_50_ determinations, the enzyme activities were measured in the presence of compound and values were estimated according to the Equation 1, where A[I] is the enzyme activity at inhibitor concentration [I] and A[0] is the enzyme activity without inhibitor.

1 $${{\rm{A}}}_{[{\rm{I}}]}={{\rm{A}}}_{[0]}\times ({\rm{1}}-\frac{[{\rm{I}}]}{[{\rm{I}}]+{{\rm{IC}}}_{{\rm{50}}}})$$

The Ki values were determined using an adapted equation for competitive inhibition (Equation 2)^20^.

2 $${\rm{v}}=\frac{{{\rm{V}}}_{{\rm{\max }}}[S]}{[{\rm{S}}]+{{\rm{K}}}_{{\rm{m}}}({\rm{1}}+\frac{[{\rm{I}}]}{{{\rm{K}}}_{{\rm{i}}}})}$$

### *In vitro* production of the PanK-7947882 and PanK-7904688 metabolite complexes

To obtain the complexes between the EthA-activated metabolite of 7947882 or of 7904688 and PanK, the enzyme (50 μM) was incubated at 37 °C with each compound (0.3 mM) in the presence of the 10 μM of the EthA monooxygenase prepared as previously described^7^. For the blank control, NADPH (0.3 mM) was omitted from the reaction mixture, in order to avoid prodrug activation. At regular intervals, aliquots were withdrawn and PanK activity was measured, to determine the enzyme inhibition levels. The activity measurements were performed as described, but with a final concentration of ATP of 0.2 mM. After 8 hours of incubation, the reaction mixture was loaded on a Ni-NTA column equilibrated in 50 mM potassium phosphate pH 7.5, washed with the same buffer to elute EthA, unbound 7947882 (or 7904688) and metabolite(s). PanK was then eluted with 250 mM imidazole in the same buffer, dialyzed against 25 mM potassium phosphate pH 7.5, 50 mM KCl and concentrated.

### Docking

The PanK protein 4BFW was prepared as described previously for PyrG^7^ for docking using the default settings of the ‘prepare protein’ protocol in Discovery Studio 4.1 (Biovia, San Diego, CA). The PanK protein (PDB ID: 4BFW) was used for docking using LibDock^21^. The protocol included 100 hotspots and docking tolerance (0.25). The FAST conformation method was also used along with steepest descent minimization with CHARMm.

## Electronic supplementary material

Supplementary Information

## References

[1] Zignol MC (2016). Twenty years of global surveillance of antituberculosis-drug resistance. N Engl J Med.

[2] WHO. Global tuberculosis report. World Health Organization (2016).

[3] Fischbach MA (2011). Combination therapies for combating antimicrobial resistance. Curr Opin Microbiol.

[4] Li K (2014). Multitarget drug discovery for tuberculosis and other infectious diseases. J Med Chem.

[5] Oldfield E, Feng X (2014). Resistance-resistant antibiotics. Trends Pharmacol Sci.

[6] Silver LL (2007). Multi-targeting by monotherapeutic antibacterials. Nat Rev Drug Discov.

[7] Mori G (2015). Thiophenecarboxamide derivatives activated by EthA kill *Mycobacterium tuberculosis* by inhibiting the CTP synthetase PyrG. Chem Biol.

[8] Ballell L (2013). Fueling open-source drug discovery: 177 small-molecule leads against tuberculosis. ChemMedChem.

[9] Esposito M (2017). A phenotypic based target screening approach delivers new antitubercular CTPs inhibitors. ACS Infect Dis..

[10] Jackowski S, Rock CO (1981). Regulation of coenzyme A biosynthesis. J Bacteriol.

[11] Evans JC (2016). Validation of CoaBC as a bactericidal target in the Coenzyme A pathway of *Mycobacterium tuberculosis*. ACS Infect Dis.

[12] Awasthy D (2010). Essentiality and functional analysis of type I and type III pantothenate kinases of *Mycobacterium tuberculosis*. Microbiology.

[13] Das S, Kumar P, Bhor V, Surolia A, Vijayan M (2006). Invariance and variability in bacterial PanK: a study based on the crystal structure of *Mycobacterium tuberculosis* PanK. Acta Crystallogr D Biol Crystallogr.

[14] Björkelid C (2013). Structural and biochemical characterization of compounds inhibiting *Mycobacterium tuberculosis* pantothenate kinase. J Biol Chem.

[15] Reddy BK (2014). Assessment of *Mycobacterium tuberculosis* pantothenate kinase vulnerability through target knockdown and mechanistically diverse inhibitors. Antimicrob Agents Chemother.

[16] Spry C, Kirk K, Saliba KJ (2008). Coenzyme A biosynthesis: an antimicrobial drug target. FEMS Microbiol Rev.

[17] Mori G, Chiarelli LR, Riccardi G, Pasca MR (2017). New prodrugs against tuberculosis. Drug Discov Today.

[18] Sambrook, J., Russel, D. W. Molecular Cloning: a laboratory manual. Cold Spring Harbor Laboratory Press, ed. 3rd (2001).

[19] Strauss E, Begley TP (2002). The antibiotic activity of N-pentylpantothenamide results from its conversion to ethyldethia-coenzyme A, a coenzyme A antimetabolite. J Biol Chem.

[20] Copeland, A. R. Enzymes: A practical introduction to structure, mechanism, and data analysis. John Wiley & Sons Inc., New York, NY, ed. 2nd (2000).

[21] Rao SN, Head MS, Kulkarni A, LaLonde JM (2007). Validation studies of the site-directed docking program LibDock. J Chem Inf Model.

