# Supplementary material for: A multitarget approach to drug discovery inhibiting Mycobacterium tuberculosis PyrG and PanK
Source: Sci Rep. 2018 Feb 16;8:3187. doi: 10.1038/s41598-018-21614-4 (PMC5816626; doi:10.1038/s41598-018-21614-4)
Supplement: Supplementary file 1 — Supplementary Information [file 41598_2018_21614_MOESM1_ESM.doc]

**Supplementary information**

**Title: A multitarget approach to drug discovery inhibiting *Mycobacterium tuberculosis* PyrG and PanK**

**Authors:**

**Laurent R. Chiarelli^1🞡^, Giorgia Mori^1🞡^, Beatrice Silvia Orena^1🞡^, Marta Esposito^1^, Thomas Lane^2,3^, Ana Luisa de Jesus Lopes Ribeiro^1,4^, Giulia Degiacomi^1^, Júlia Zemanová^5^, Sára Szádocka^5^, Stanislav Huszár^5^, Zuzana Palčeková^5^, Marcello Manfredi^6^, Fabio Gosetti^6^, Joël Lelièvre^7^, Lluis Ballell^7^, Elena Kazakova^8^, Vadim Makarov^8^, Emilio Marengo^6^, Katarina Mikusova^5^, Stewart T. Cole^9^, Giovanna Riccardi^1^, Sean Ekins^10,2^, Maria Rosalia Pasca^1*^**

^1^Department of Biology and Biotechnology “Lazzaro Spallanzani”, University of Pavia, Pavia, Italy; ^2^Collaborations Pharmaceuticals, Inc., Main Campus Drive, Lab 3510 Raleigh, NC 27606; ^3^Molecular and Cellular Biophysics Program, Department of Biochemistry and Biophysics, University of North Carolina, Chapel Hill, NC 27599, USA; ^4^Centro de Biologia Molecular "Severo Ochoa", Universidad Autónoma de Madrid, Madrid, Spain; ^5^Department of Biochemistry, Faculty of Natural Sciences, Comenius University in Bratislava, Bratislava, Slovakia; ^6^Department of Sciences and Technological Innovation, University of Piemonte Orientale, Alessandria, Italy; ^7^Diseases of the Developing World, GlaxoSmithKline, Tres Cantos, Madrid, Spain; ^8^Lab for Biomedicinal Chemistry, Bach Institute of Biochemistry, Research Center of Biotechnology of the Russian Academy of Sciences, Moscow 119071, Russia; ^9^Global Health Institute, Ecole Polytechnique Fédérale de Lausanne (EPFL), Lausanne, CH-1015, Switzerland; ^10^Collaborative Drug Discovery, Inc. Burlingame, CA, USA.

|  | **Pantothenate** | | | **ATP** | | |
| --- | --- | --- | --- | --- | --- | --- |
|  | *k*_cat_  (s^-1^) | *K*_m_  (mM) | *k*_cat_ / *K*_m_  ( s^-1^ mM^-1^) | *k*_cat_  (s^-1^) | *K*_m_  (mM) | *k*_cat_ */ K*_m_  ( s^-1^ mM^-1^) |
| **Wild type PanK** | 6.3±0.3 | 0.28±0.03 | 22.5±2.7 | 6.1±0.2 | 0.19±0.02 | 32.1±1.7 |
| **Q207R mutant PanK** | 1.8±0.2 | 0.40±0.08 | 4.5±0.7 | 2.0±0.1 | 3.56±0.36 | 0.5±0.4 |

**Table S1. Kinetic constants of the *M. tuberculosis* recombinant wild type and mutant PanK enzymes.**

| **Primers** | **Sequence (5’-3’)** | **PCR product (bp)** | **Purpose** |
| --- | --- | --- | --- |
| CoaAseqFor | GCACTACGACATCATCCC | 231 | Sequencing of *coaA* |
| CoaAseqRev | TAGTGGTGGAAGTGTGATT |
| CoaApET28For | TTTggatccATGTCGCGGCTTA (BamHI) | 957 | Cloning of *coaA* in pET-28a |
| CoaApET28Rev | TTTaagcttTTACAGCTTGCGCAG (HindIII) |

**Table S2. Oligonucleotide primers used in this study.**

**Figure S1. Examination of effects of GSK PyrG inhibitors on *M. tuberculosis* H37Ra cells by [^14^C]-acetate metabolic labeling.**

The effects of the GSK PyrG inhibitors on the mycobacterial lipids were assessed through metabolic labelling with [^14^C]-acetate as previously reported^1^, slightly modified. Briefly, *M. tuberculosis* H37Ra was grown statically in 7H9 medium supplemented with 10% ADC and 0.05% Tween 80 until OD600~0.26. The compounds were added at 4x MIC; the control drug, BTZ043, was added at 0.2 μg/ml. After 24 hr drug treatment, [^14^C]-acetate (American Radiolabeled Chemicals, specific activity 106 mCi/mmol) was added to a final concentration 0.5 μCi/ml, and the incubation continued for next three hours. Growth inhibition at harvest was evaluated by measuring of OD_600_ and reached for GSK1570606A (606) – 37%, for GSK920684A (684) – 26%, for GSK735826A (826) -66%, for 11426026 – 83% and for BTZ043 – 23%. Bacteria were then subjected to extractions and fractionation as described^1^. The final organic phase was dried and dissolved in Solvent I in the ratio 75 μl/OD600 0.5/2 ml of the culture. 5 μl aliquots of the lipid extracts were analyzed by TLC on Silica gel plates (Merck) in Solvent I: CHCl_3_/CH_3_OH/NH_4_OH/H_2_O (65:25:0.5:4); Solvent II CHCl_3_/CH_3_OH/H_2_O (20:4:0.5); and Solvent III: petroleum ether/ethyl acetate (98:2; 3 times). After chromatography, the plates were exposed to autoradiography film (BioMax MR) at -80°C for 7 days. TDM, trehalosedimycolates; TMM, trehalosemonomycolates; PE, phosphatidylethanolamine; CL, cardiolipin; PI, phosphatidylinositol; PIM, phosphatidylinositol mannosides; TAG, triacylglycerol.

**REFERENCE**

1. Esposito, M., *et al*. A phenotypic based target screening approach delivers new antitubercular CTPs inhibitors. ACS Infect Dis. **3**, 428-437. doi: 10.1021/acsinfecdis.7b00006 (2017).
